# Why do preschoolers perpetuate inequalities? Theoretical perspectives on inequity preferences in the face of emerging concerns for equality

**DOI:** 10.1016/j.dr.2020.100933

**Published:** 2020-12

**Authors:** Markus Paulus, Samuel Essler

**Affiliations:** Ludwig-Maximilians-Universität München, Munich, Germany

**Keywords:** Sharing behavior, Resource allocation, Fairness, Unfairness, Equality, Preschoolers

## Abstract

Recent research has shown that preschool children tend to preferentially allocate resources to rich than to poor others. The findings that young children tend to perpetuate inequalities are puzzling given classical developmental theories that largely focused on the emergence of equality and equity in childhood. In this review, we first sketch the early ontogeny of fairness concerns before providing an overview on studies reporting perpetuation of inequality in young children. We review four classical theories (Piaget, Kohlberg, Damon, Social Domain Theory) and discuss how they would account for this phenomenon. We then introduce four recent theoretical models that directly speak to the underlying psychological processes; the affective preference model, the reciprocity-based strategic model, the numerical matching model, and the normative model. We highlight the key tenets of each model, their relation to other developmental processes, and the strength of the empirical evidence. From each model, we derive specific hypotheses. Finally, in an integrative section we discuss how the models might relate to each other, highlight connections to other research areas, and present avenues for future research.

## Highlights

• Recent research has shown that preschool children tend to perpetuate inequalities.
• We review classical and current theoretical accounts that attempt to explain this tendency.
• Four current theoretical models are suggested that highlight different perceptual, affective, and cognitive processes.
• We evaluate the empirical strength of each model and derive hypotheses for future work.

## Introduction

The unequal distribution of resources, that is, wealth and poverty, are ubiquitous characteristics of human societies. The asymmetry in resource possessions constitutes a key factor in structuring societies and greatly impacts individuals’ opportunities and welfare. While all societies know efforts that aim at reducing inequalities, humanity also experiences tendencies that maintain and bolster social inequalities. That is, while people report equality to be a main concern (Dawes et al., 2007, Turiel, 2002), it has also been observed that they accept and perpetuate inequality (e.g., Boyer and Petersen, 2018, Piff et al., 2018, Starmans et al., 2017).

More specifically, an abundance of studies with adults makes a strong case for a pronounced concern for equality and inequality aversion in humans. For example, people tend to allocate outcomes of a group effort equally between participants (Harris & Joyce, 1980), have distinct negative emotions towards advantaged others in a context of inequality and attempt to decrease the inequality even at a personal cost (Dawes et al., 2007), and refuse to make reciprocal decisions when they increase inequality (Xiao & Bicchieri, 2010). Yet, another strand of research shows that adults sometimes both accept inequalities and actively perpetuate them. This is true when they view inequalities as fair and justified (Starmans et al., 2017). For example, Norton and Ariely (2011) report Americans’ rejection of the current distribution of wealth across political parties, but also find that participants asked to construct an ideal distribution of wealth opt for an unequal scenario (though much more equal than the current distribution). Similarly, adults universally judge the pay gap between CEOs and unskilled workers as too high, but consider a pay ratio of about 7:1 as justified and fair, rather than equal pay (Kiatpongsan & Norton, 2014). Notably, the tendency to perpetuate inequality is also present beyond a consideration of inequality as being fair. Ho et al. (2015) present a new conceptualization of social dominance orientation. Especially noteworthy is the subscale SDQ-E (SDQ-Egalitarianism) that measures preferences for group based inequalities independent of interrelated fairness and inequality concerns. Moreover, there are notions of differing psychological dynamics in upper-class and lower-class individuals, that have been proposed to result in reproductions of inequalities (Piff, Kraus, & Keltner, 2018). While upper-class individuals concentrate on self-gain and an increase of power, lower-class individuals show less self-focus in that regard, leading to exacerbated economic inequalities. These dynamics highlight notions of actively perpetuating inequalities in addition to tendencies that speak more to the mere acceptance of inequality (e.g., Ho et al., 2015, Norton and Ariely, 2011).

Thus, we see a field of tension between the perpetuation and rejection of inequality in humans. A plentitude of psychological mechanisms for the perpetuation of inequalities have been discussed, inter alia system justification tendencies (e.g., Jost & Banaji, 1994), folk-economic beliefs about economic inequalities (e.g., Boyer & Petersen, 2018), and trait-based approaches (e.g., Ho et al., 2015). Overall, both concerns (that is, concerns for equality and tendencies to perpetuate inequality) could be among the driving forces in societal decision-making. Historically, the ongoing seesaw between these two might explain political and societal change. In light of this longstanding opposing dynamic, it would be interesting to look back into early ontogeny to retrace the emergence of humans’ concerns for equality and perpetuation of inequality.

## The development of concerns for equality and fairness

For many decades developmental scholars had focused on the early origins and development of fairness principles (Damon, 1977, Piaget, 1932). One core area of developmental research explores when and how equality becomes a key concern for young children (e.g., Blake and McAuliffe, 2011, Carpendale et al., 2013, Damon, 1977, DeJesus et al., 2014, Elenbaas et al., 2016, Smith et al., 2013, Ulber et al., 2015). Moreover, it has been explored when and under which circumstances children realize or endorse more complex fairness principles such as merit or need (e.g., Baumard et al., 2012, Elenbaas, 2019, Kanngiesser and Warneken, 2012, Malti et al., 2016, Wörle and Paulus, 2018).

By and large, the wealth of recent research suggests that at least by 3–4 years, children start to explicitly appreciate equality. This is evidenced in their ideas of how one should distribute resources (Smith et al., 2013), their expectations how others will distribute resources (DeJesus et al., 2014), and their active enforcement of equal sharing towards third-parties (Rakoczy, Kaufmann, & Lohse, 2016). This emphasis on equality is foreshadowed by so-called implicit measures with even younger children that indicate some nascent sensitivity for equality (for review see Sommerville & Enright, 2018). In third-party resource allocation tasks – that is, when children themselves are not possible recipients, but have to distribute resources among two others – they often tend to realize an equal split (e.g., Baumard et al., 2012, Huppert et al., 2019), at least if there is no other difference between the two recipients. The picture becomes more blurry when considering their own sharing behavior – that is, tasks in which children have to give up own resources or are themselves potential recipients of the resources. Here, some studies reported that already preschool children (around 3–5 years) tend to share equally (Blake and Rand, 2010, Paulus and Rosal-Grifoll, 2017), particularly when having engaged in collaborative activity (Warneken, Lohse, Melis, & Tomasello, 2011). Other studies reported that it is by middle childhood (around 7–10 years) that children realize equal distributions between themselves and another person (Christner et al., 2020, Samek et al., 2020, Smith et al., 2013). With increasing age, children not only consider numerical equality, but also the value and type of ressources, and tend to realize equality on a qualitative level (Chernyak and Sobel, 2016, Sheskin et al., 2016). In addition, by middle childhood, they also tend to accept costs for themselves and reject advantageous offers in order to realize equality (Blake and McAuliffe, 2011, Shaw and Olson, 2012), although this pattern seems to be mostly prevalent across Western cultures rather than constituting an universal trend (Blake et al., 2015, House et al., 2013). This variability in age could probably be explained by different task demands that seem to differ in terms of, for example, whether children already obtained the entire set of resources and now have to give up some of them for the other (Blake & Rand, 2010) or whether they instruct an experimenter how to distribute a set of items that not yet belonged to them (Paulus, 2014). Overall, despite some variability in tasks and contexts, we see an increasing concern for equality across early and middle childhood.

On a theoretical level, the increased realization of equality has been related to developmental increases in self-control and emotion regulation capacities (Eisenberg, 2000), theory-of-mind (Yu, Zhu, & Leslie, 2016), emerging moral emotions (Malti & Krettenauer, 2013) as for example evident in an increased anticipation of negative feelings when not engaging in fair behavior (Christner et al., 2020), and an increased sense of obligation to moral norms (Nunner-Winkler, 2007).

In the course of development, more complex fairness principles (such as merit or need) start to play a greater role (Damon, 1977). For example, a recent cross-cultural multi-site study demonstrates an universal age-related shift from equality to equity between early childhood and middle childhood (Huppert et al., 2019) suggesting that simple forms of fairness become complemented by more complex fairness considerations in the course of middle childhood (but see also Elenbaas, 2019). Overall, developmental research suggests an emergence of concerns with equality in early childhood that is mostly prevalent when children consider how resources should be distributed and when they distribute resources between two neutral others. Sharing own resources seems to be more demanding and the realization of costly equal sharing is mostly evident in older children.

## Young children perpetuate inequality

Surprisingly, in the past years developmental research has revealed a consistent, yet puzzling finding. Across several experimental designs, studies reported that (a subgroup of) preschool children show a tendency to prefer rich others, and distribute more resources to them than to poor individuals (Essler et al., 2020, Kenward et al., 2015, Li et al., 2014, Olson et al., 2011, Paulus, 2014, Paulus, 2020, Rizzo and Killen, 2016; for an overview see Table 1). That is, a non-neglectable number of young preschool children seem to perpetuate inequality - rather than engaging in either equality or idiosyncratic sharing decisions – and this tendency does not seem to be based on fairness considerations (e.g., considerations of merit or need). Rather, it seems as if the mere difference in preexisting distributions leads to the perpetuation of inequality. This phenomenon is the focus of this paper.

**Table 1:** Recent key studies reporting perpetuation of inequalities in children.

Study	Age Group	Context of Inequality	Method (e.g., first or third person)	Findings Concerning Perpetuation of Inequality
Essler et al., 2020	3–5-year olds	Economic inequality between rich and poor other	• Third party resource allocation between a poor and a rich character with necessary vs. luxury resources (between-subjects)	• Inequitable allocators (about one third of sample) largely matched the inequality between the rich and the poor other in their allocations • Inequitable allocators preferred inequitable over equal and equitable allocation strategies
Kenward et al., 2015, Experiment 1	4-, 6-, and 8-year olds	Economic inequality between a rich (could reciprocate) and a poor (could not reciprocate) other	• First party resource sharing task with rich or poor experimenter (rich experimenter had greater possibility to reciprocate during the game than the poor one)	• Across age groups, children favored the rich recipient, who was able to reciprocate, in their resource allocations
Kenward et al., 2015, Experiment 2	4-year olds	Economic inequality between a rich (could reciprocate) and a poor (could not reciprocate) other	• First party resource sharing task with rich or poor experimenter (rich one had greater possibility to reciprocate during the game and stated intention to reciprocate)	• Children favored a rich recipient, who was able to reciprocate and stated the intention to reciprocate • But children ceased to favor the rich recipient when he did not act on his intention to reciprocate
Olson et al. (2011), Experiment 1	4.5–11.5-year olds	Economic inequality in resources between two social groups	• Inequality between Blacks and Whites → third party resource distribution of 3 cookies	• The majority of younger children (4.5–7.5) perpetuated the inequality • The majority of older children (7.5–11.5) rectified the inequality
Olson et al. (2011), Experiment 2	4.5–11.5-year olds	Economic inequality in resources between two social groups	• Inequality between Asians and Whites → third party resource distribution of 3 cookies	• Younger (4.5–7.5) and older (7.5–11.5) children tended to perpetuate the inequality
Olson et al. (2011), Experiment 3	4.5–11.5-year olds	Economic inequality in resources between two social groups	• Inequality between novel groups (green vs orange) instead of racial groups → third party resource distribution of 3 cookies	• Younger (4.5–7.5) and older (7.5–11.5) children tended to perpetuate the inequality
Paulus (2014), Experiment 1	3-year olds and 5-year olds	Economic inequality between a rich and a poor other	• First party sharing task with a rich and a poor other	• 3-year-olds acted less selfishly when sharing with the rich other than the poor other, showing a trend to perpetuate the inequality (non-significant tendency)
Paulus (2020)	1.5-year olds	Inequality in need of help: needy and non-needy other	• 6 helping tasks in which one experimenter was in need of help (needy other) and the other one was not (non-needy other)	• Young children helped non-needy other over needy other, showing a trend to increase the disparity in need (non-significant tendency)
Paulus and Rosal-Grifoll (2017)	3–6-year olds with ASD	Economic inequality between a rich and a poor other	• First party sharing task with 10 stickers to distribute between participants themselves and a sticker-rich as well as a sticker-poor recipient	• The stronger the ASD symptoms and the cognitively weaker the ASD children, the more they allocated to rich over poor other
Elenbaas and Killen (2016)	3–6-year olds	Economic inequality between two groups	• Third party expectations: Children give expectations about how members of advantaged and disadvantaged groups would evaluate the inequality, which group they would prefer, and how they would allocate resources between the groups	• If allocators from advantaged groups evaluate inequality positively, they are expected to perpetuate it
Li et al. (2014)	4–5-year olds	Economic inequality between rich and poor other	• Preference rating & third party resource allocation	• Children liked advantaged recipient more and allocated more to the advantaged recipient if they were not aware anymore that he is advantaged • Participants allocated more to the disadvantaged recipient when they were aware of the disadvantage during the allocation

In some studies, these effects are clear and strong on a group level (e.g., Kenward et al., 2015, Li et al., 2014), whereas other studies reported this effect only for subgroups (e.g., Essler et al., 2020). Importantly, this trend has been reported across different study designs and tasks, ranging from first-person sharing contexts in which children can share own resources with rich and poor others (e.g., Kenward et al., 2015) to third-person resource allocation tasks in which children distribute resource between poor and rich other (e.g., Essler et al., 2020, Olson et al., 2011). In addition, this phenomenon has been found in studies which provide children with an even number of resources to share or distribute (Essler et al., 2020, Paulus, 2014, Rizzo and Killen, 2016) as well as in studies providing children with an odd number of resources to share or distribute (Kenward et al., 2015, Li et al., 2014, Olson et al., 2011). Notably, the majority of these studies go beyond a mere acceptance of inequality and provide evidence for active perpetuation of inequality (e.g., Essler et al., 2020, Li et al., 2014, Olson et al., 2011, Kenward et al., 2015).

For example, Kenward et al. (2015) presented 4-, 6-, and 8-year-old children with a game context in which they could distribute resources between a rich recipient, who was able to reciprocate, and a poor recipient, who was not able to reciprocate. Across age groups, children favored the rich recipient, but older children tended to distribute resources more equally. Interestingly, younger children gave more to a rich other when the other could reciprocate, but ceased to do so when the rich either could not reciprocate anymore or disappointed children’s expectations of reciprocity. Moreover, outside of a context that allowed for reciprocity, Essler et al. (2020) reported that in a sample of 3- to 5-year-old children, about one third (mostly younger preschoolers) allocated more to a rich than a poor other and also preferred inequitable over equal and equitable allocation strategies. With increasing age, children tended to allocate more resources to the poor recipient. The tendency to allocate resources to rich others has been related to cognitive functioning: a study with 3- to 6-year-old preschool children with autism spectrum disorders (ASD) found that lower cognitive abilities (as assessed by a working memory task) and more severe ASD symptoms were related to an increased generosity towards a rich other (Paulus & Rosal-Grifoll, 2017). Another study with 4–5-year-old children reported that while participants were initially more likely to rather distribute resources to a poor than to a rich, they (in a later phase) favored the rich recipient when they had forgotten about the initial distribution (Li et al., 2014). Moreover, this work is complemented by findings that young children favor a dominant over a subordinate other in resource distribution contexts (Charafeddine et al., 2016). Notably, under some circumstances preschool children also expect others to perpetuate an inequality. If they are confronted with an unequal resource distribution between two groups, they expect allocators from the advantaged group to increase the inequality, if they evaluate the resource disparity positively (Elenbaas & Killen, 2016).

Given the parallel sets of findings that young children appreciate equality (Smith et al., 2013) and even enforce equal distributions from third-parties (Rakoczy et al., 2016), this set of findings is theoretically challenging. That is, these findings are puzzling given longstanding claims that – before starting to appreciate equality – young children distribute resources unsystematically and mostly follow subjective preferences (Piaget, 1932). Given the field’s long-standing theoretical focus on the emergence of fairness, young children’s perpetuation of inequality has remained theoretically underexplored and is currently not well understood.

Moreover, they are interesting in light of the above mentioned debate on the nature of humans’ perpetuation of inequality (Boyer and Petersen, 2018, Starmans et al., 2017) as they could point to an early psychological basis of this tendency. Why and under what circumstances do young children show these distribution behaviors? What is their psychological basis? Given that the discussion of these findings is scattered in the literature, the following sections will introduce several theoretical accounts that could account for these findings. We will first shortly consider classical approaches that might speak to this issue and then expand on current theoretical accounts. We evaluate the strength of these recent accounts based on empirical findings and in light of their ability to explain developmental changes. Finally, we conclude with an outlook and avenues for further research.

## Classical views on the ontogenetic emergence of equality concerns

Notably, although classical developmental theories have mostly focused on the emergence of a concern for equality, they also offer conceptual frameworks that allow for an understanding of young children’s perpetuation of inequality. This section therefore aims at exploring how classical theories would explain young children’s perpetuation of inequality and at preparing the ground for the subsequently discussed more recent approaches. In the following we will first focus on Piaget's (1932) theory that has had a major impact on the field and whose key tenets are still shared by current accounts (e.g., Carpendale et al., 2013, Hammond, 2014). We will then shortly consider the theoretical frameworks by Kohlberg, 1976, Kohlberg, 1986, Damon, 1977 before turning to Turiel's (1983) foundation of the social domain theoretical (SDT) approach that builds a bridge to contemporary work (e.g., Smetana, 2013).

For Piaget (1932), moral development can be – by and large – described as a progression along the lines from 1) idiosyncratic behavioral rituals to 2) egocentric behavior and constraint-based rule-following, and 3) cooperative behavior based on mutual respect. Moral development is rooted in preverbal idiosyncratic rituals in which infants and toddlers repeat actions and structure their behavior with objects or their interactions with others by building action schemes (e.g., building towers with bricks). This regularity in behavior does not contain a sense of duty, but these ritualized interactions are building blocks that could explain the presence of simple prosocial behaviors such as instrumental helping and single instances of giving/sharing (Hammond, 2014, Paulus, 2014). Concerning children’s behavior in games and other rule-based joint activities, this first phase is followed by an egocentric phase in which children have the desire to play like others and to be part of social interactions with peers, and hence imitate their rules and behavior, but nonetheless they play by their own. That is, their play follows each own’s logic, is not supervised by others and not standardized. In the last phase(s), children start to cooperatively organize their games, discuss the rules, and eventually develop an intellectual interest in the rules per se and in a codification of rules. A similar logic is evident in children’s rule consciousness in which young children follow own idiosyncratic routines and external rules without a reflection on rules. Children then develop a sense of obligation to appreciate and stick to rules based on respect for authorities (adults or older children). This so-called heteronomous orientation emerges out of social interactions that are characterized by constraint and that are prevalent in early childhood. Finally, this is followed by an understanding of the negotiability of rules and their origins in mutual consent. This autonomous morality emerges in social interactions with equals (usually peers) that are characterized by cooperativity.

Finally, the same trend from rather heteronomous to autonomous views is present in children’s concept of justice. In a first phase, children do not differentiate between just and dutiful as well as unjust and disobedient. Justice is based on the rules of adult authority. This is followed by strong egalitarianism, which is eventually qualified by an appreciation of equity and a consideration of contexts. Like the development of rules, a real appreciation of the spirit of justice (and not only the literal laws of rules of distribution) requires forms of cooperative social interactions and mutual respect among equals.

It should be noted that Piaget (1932) stated that in moral development, there are no clearly circumscribable stages (see also Carpendale, 2000). That is, while there is a certain logic of development in which previously acquired skills form the basis for further developmental achievements, there are no unitary and clearly delineated stages. Rather, there are fluent passages and different orientations that could be present in different contexts (most notably, constraint-based morality in interactions with authority figures and cooperation-based morality in interactions with peers). Moreover, action is supposed to precede thought so that principles of justice will be observable in children’s behavior before they start to cognitively reflect on it.

How could we explain the active perpetuation of inequality from a Piagetian point of view? His theory seems to offer two possibilities. Piaget’s focus on idiosyncratic rituals invites the hypothesis that children acquired behavioral routines in which they match items to already preexisting distributions. This should be particularly true for very young children who have not yet developed rule consciousness or whose appreciation of rules do not allow to solve the problem. This thought antedates the numerical matching hypothesis as outlined below. Second, related to the first phase of the development of a concept of justice, children could accept and even perpetuate a preexisting inequality insofar as it is endorsed by adult authority. This consideration overlaps with the normative model.

This brings us to the second classical account, Kohlberg, 1976, Kohlberg, 1986 theory, that differed to some extent from Piaget as it followed a strict stage-wise logic and as it put priority on children’s reasoning rather than their action. Kohlberg took the approach of identifying stages of moral development from the at that time common standard interpretation of Piaget (Carpendale, 2009). He provided a framework on development within the moral domain defined as the domain of “justice judgments and reasoning” (Kohlberg, 1986, p. 486). He distinguished different aspects of justice, with one of it – most relevant for the purpose of this manuscript – concerning distributive justice. In his view, moral development unfolds over 3 stages (consisting of two phases each) that can be understood as progressive decentration and expansion of social perspectives (Habermas, 1983). Stage 1, being described as heteronomous morality, is characterized by strict equality. Equity concerns are not considered. Similar to Piaget (1932), norms and rules are applied in a literal manner and the child can be seen as a moral realist. Most interesting for our purpose, Kohlberg (1986) stated that unequal distributions “can be acceptable if to persons of a less valued category” (p. 491). That is, under some circumstances children’s strict appreciation of equality pertains only to members of a particular category, but not to others (Kohlberg, 1986). Although not fully spelled out, this suggests that in Kohlberg’s view the perpetuation of inequality can happen when there is a categorical distinction between recipients. This may hint to an implicit conviction that unequal things do not need to be treated equally, although this was, to our knowledge, not systematically explored by Kohlberg. A further implication is that endorsement of equality and perpetuation of inequality could develop and exist in parallel, and might not form a developmental sequence in early childhood.

In a similar vein, Damon (1977) suggested a developmental sequence of six levels (from 0-A to 2-B) in children’s conceptions of positive justice. The progression along these levels consists of a disentanglement of initially confounded concepts (e.g, own views and the views of others). Most interesting for our purpose are the first three levels (0-A, 0-B, 1-A) as they give a more differentiated view on early childhood as did Kohlberg’s approach. At level 0-A (mostly at 4 years), children confound justice and fairness with their own wishes, arguing that an action is fair as they prefer the consequences of this action. This level is characterized by an assimilation of the world and others’ views on own wishes. Children strive for pleasant experiences for themselves or those who are emotionally close to self (e.g. family and friends) or with whom they identify otherwise (e.g., Gender, Age, Ingroup). At level 0-B (mostly at 4–5 years), children rely on quasi-objective, but irrelevant characteristics in order to justify their preferences (e.g., “I should receive more because I am a boy.”). Eventually, Level 1-A (mostly by 5 years) is characterized by strict equality. That is, perpetuation of inequality is supposed to be a developmentally earlier phenomenon than an appreciation of equality. A further implication of this view is that in a resource allocation context young children might perpetuate inequality if the rich other is similar to or emotionally close to themselves. This account seems to foreshadow some of the thoughts developed in the affective preference model.

A different approach was taken by Turiel (1983). He abandoned the idea of a stage-wise ontogenetic progression in moral development that can be described as a process of increasing differentiation of initially intertwined or globally fused concepts of the social world. Rather, he proposed that from early on, young children construct different domains of social knowledge and social rules. This constructive process is based on children’s experiences in social interactions (Dahl, 2018, Smetana, 1983). Most centrally, he distinguishes between a moral domain and a social-conventional domain. The moral domain is defined by considerations of fairness and welfare that are noncontingent, unalterable, and independent of consensus. In his view, young children endorse principles of fairness such as equality and equity, and oppose immoral actions even when permitted by an authority. From a SDT perspective, a perpetuation of inequality is difficult to explain. To us, it seems that it could only be explained by extraneous factors overriding genuine moral judgment or by children having genuine moral reasons to perpetuate inequality (e.g., when they were to believe that perpetuating inequality would increase the welfare of the involved parties). One could speculate that the latter explanation shares some ideas with a normative model (as outlined below).

Taken together, the classical views have largely focused on explaining the emergence of morality and fairness, most notably equality and equity. Nonetheless, they give important hints and allow for theory-grounded considerations on why young children perpetuate inequality. In addition, these lines of thought preempt the theoretical considerations that have been put forward by current theoretical accounts

## Current theoretical accounts

Given recent findings on perpetuation of inequality in young children, theoretical considerations have been put forward that aim to account for that. A closer reading of the current literature allows for an identification of four different theoretical models. While some aspects of these accounts are foreshadowed by classical theories, they all provide an advancement of theorizing on the specific psychological mechanisms underlying the perpetuation of inequity. Although each account aims to explain the range of phenomena, it should be noted that the accounts are not necessarily mutually exclusive. In the following, we will introduce and discuss each of these accounts in turn.

### Affective preference model

The first account highlights affective factors involved in resource allocation decisions. More precisely, this model suggests that young children have an affective preference for rich over poor others that might lead children to favor rich over poor recipients in their allocations. This affective preference might be related to a general preference for lucky over unlucky others in young children (Olson, Banaji, Dweck, & Spelke, 2006). That is, young children might perceive of wealth as a factor that is inherently connected to positive emotional states (e.g., well-being, happiness). As positive emotional states and positive others are more attractive than negative states and negative others, children might feel drawn towards preferentially interacting with lucky and rich others. Consequently, they are also more likely to distribute more resources to them. This could also explain why young children favor individuals and groups that have a higher socioeconomic status (Horwitz, Shutts, & Olson, 2014) or are generally more lucky (Olson et al., 2006). In addition, children themselves might have received more resources from rich others. If they repeatedly make this positive experience with rich individuals, they might generalize it to all members of this class and see them positively. Thus, there are several routes how children might develop affective preferences for rich persons.

From a developmental perspective, this account has clear plausibility. It ties in with views that young children’s behavior is mostly guided by affective and behavioral preferences, and becomes subject to cognitive control and reflection in the course of development (Allen and Bickhard, 2018, Moore, 2009). Indeed, recent accounts stress the presence of a simple approach-avoidance system as a basis of children’s moral development (Cowell & Decety, 2015). Moreover, it relates well to findings that young children mainly differentiate between positively and negatively valenced emotions (Harter & Rumbaugh-Whitesell, 1990) before developing a more nuanced understanding of their social world.

The strongest evidence for this account comes from a study by Li et al. (2014). In this study, older preschool children (4–5 years) allocated more resources to a poor other while simultaneously rating the rich other more positively. When introducing a short time delay during which children forgot the initial distribution of resources, children gave more resources to the rich other. This indicates that their affective preference for the rich other remained and guided their resource allocation decisions. Moreover, the tendency to characterize poor others with more negative attributes and, conversely, see rich others more positively prevails across life (e.g., Mistry, Brown, White, Chow, & Gillen-O’Neel, 2015) and shows itself in a variety of stereotypes (e.g., Williams, 2009). In general, the affective preference model is very appealing as the phenomenon seems to be more pronounced in young children (3–4 years) and seems to decline in the following years (e.g., Kenward et al., 2015; see also Charafeddine et al., 2016). Given that cognitive abilities increase with age and assuming that young children’s behavior is more strongly driven by affective factors than by cognitive reflection, it seems reasonable to assume that young children’s unequal sharing is based on affective preferences. Yet, this account is difficult to align with findings by Kenward et al. (2015) who reported that children gave more to a rich other, when it was strategically reasonable, but preferred to allocate to the poor in another context.

Overall, this model suggests a dynamic interplay of affective and cognitive processes. It assumes that the processes leading to a perpetuation of inequality (affective preference) and to a realization of equality or equity (cognitive processes) exist in parallel. This implicates that there is rivalry between affective and cognitive processes, and that it depends on contextual factors which one will prevail. Assuming that the affective preferences for rich others are based on a simpler psychological process whereas the cognitive view that poor others should receive more than rich others represents a cognitively more demanding strategy, this model could also explain why perpetuation of inequality is often rather found in young preschool children and gives way to equality and equity later in development. From a life-span perspective, affective preferences might also play a role in older children although with increasing age cognitive justifications for inequality (e.g., based on skill or choice) start to prevail (Karniol, 1985, Sigelman, 2012; see also Flanagan et al., 2014). In adults, emotions towards the rich might also be affected by differing beliefs about the causes of inequality (e.g., Ho et al., 2015, Jost and Banaji, 1994). Thus, affective dynamics likely appear much more contextualized (depending on beliefs, traits etc.) in adults than in children, yet they still seem to be present.

This model allows for the hypothesis that the preferential treatment of rich others should be more enhanced in children that have not developed a normative stance that one should rectify inequalities (e.g., Wörle & Paulus, 2018). Moreover, the reference to an interplay between affective and cognitive processes reminds the reader on dual-process accounts of human cognition according to which the dynamic antagonism between an intuitive affective system and a more deliberate cognitive system explain behavior. An interesting alternative is provided by the application of fuzzy trace theory to moral development (Kogut and Slovic, 2016, Reyna and Casillas, 2009). It is possible that young children’s verbatim representation (of the equity principle) guides their initial allocation of resource to the poor whereas a gist-like representation of the recipients (indicated by children’s seemingly oblivion of details) guided their subsequent allocation to the rich. Finally, reconsidering Damon's (1977) framework it would be interesting to explore whether the affective tagging is particularly strong for individuals with whom children can identify to a greater degree. Research on young children’s ingroup biases even for minimal groups (e.g., Dunham, Baron, & Carey, 2011) hints to this possibility.

### Reciprocity-based strategic model

A second account emphasizes the role of cognitive and strategic factors and in particular children’s expectations towards the future behavior of the recipient in their preference for the wealthy other. More precisely, it is possible that children evaluate the initial resource distribution and realize that the rich other will be more likely to reciprocate their generosity in the future. Thus, if children’s resource allocation is mainly motivated by self-interest, they are better off in distributing resources to the rich other than to the poor other, as the rich other has more to offer than the poor one in a future exchange. This account relates well to recent findings. In a series of studies, Ahl and colleagues demonstrated that 4- to 10-year-old children predict that a rich other will be more likely to share than a poor other (Ahl and Dunham, 2019, Ahl et al., 2019). Moreover, preschool children expect others to reciprocate the benefits they have received (Paulus, 2016). From a strategic point of view, it thus makes sense to rather share with rich others.

Notably, this model ties in with a large set of recent developmental studies that demonstrated that preschool children engage in a variety of reciprocal behaviors (e.g., Beeler-Duden and Vaish, 2020, Chernyak et al., 2019, Wörle et al., 2020) and expect others to behave reciprocally (Olson & Spelke, 2008). Moreover, by about 4 years, strategic considerations start to play a greater role in sharing behavior. Children are more generous if the recipient has the possibility or intention to reciprocate in a subsequent phase (Sebastián-Enesco and Warneken, 2015, Xiong et al., 2016). The emergence of strategic considerations can be related to developments in children’s appreciation of time and future states (e.g., Prabhakar and Hudson, 2014, Suddendorf et al., 2011), in their planning abilities (e.g., Martin-Ordas, 2018, McCormack and Atance, 2011), and in their social understanding (Takagishi, Kameshima, Schug, Koizumi, & Yamagishi, 2010). Notably, while an increase in social understanding is related to enhanced sharing (Imuta, Henry, Slaughter, Selcuk, & Ruffman, 2016), free sharing in a context without social consequences of non-sharing seems to be negatively related to Theory-of-Mind (Cowell, Samek, List, & Decety, 2015), pointing to strategic considerations in preschoolers’ sharing. In addition, from a life-span perspective, one could speculate that this seems like a mechanism that could be relevant for explaining adolescents’ and adults’ preferential behavior towards rich or advantaged others in general. Indeed, although the field seems to lack conclusive empirical research, it has been hypothesized that strategic forms of sharing exacerbate inequalities (Piff et al., 2018).

Direct evidence comes from the above mentioned study in which 4- to 8-year-old children were playing a three-player game with two others in which windfall resources had to be shared with one of the other players (Kenward et al., 2015). One of the players was resource rich, whereas the other one had no resources (and could thus not pay back). During the game, children were more likely to share with the rich other than with the poor individual. Yet, when children had to leave the game (and no future reciprocation was thus possible), they left their remaining resources with the poor individual. This pattern suggests that their previous resource allocation decisions were driven by strategic considerations. Yet, this model cannot account for a preference for rich others even in the absence of memories who of two persons was actually richer (Li et al., 2014).

An interesting implication of the strategic model is that perpetuation of inequality is cognitively more effortful than the application of an equality rule (for neurocognitive results and debate see also Meidenbauer et al., 2018, Pletti and Paulus, 2020). One could thus hypothesize a developmental sequence according to which perpetuation of inequality should be more enhanced in older children, particularly when reciprocation is possible. This also relates to findings that fairness-based inequality (that is, inequality based on merit or need) seems to emerge later than equality (Elenbaas et al., 2016, Malti et al., 2016, Wörle and Paulus, 2018). In addition, given the cognitive prerequisites of strategic behavior, this account would hypothesize that perpetuation of inequality is more pronounced when cognitive control and strategical thinking is more developed.

### Numerical matching model

A third account focuses on rather perceptual factors that could give rise to children’s distribution decisions. This account has deep roots in developmental theorizing and relates to the proposal that one strategy young children are able to use is matching based on perceptual similarity (Piaget & Szeminska, 1952). On the one hand, the matching process might describe a routine or behavioral scheme that originates in young children’s exploration and play behavior (cf. Piaget, 1932). On the other hand, the numerically differing amounts of possessions children are exposed to prior to their own distributions might produce an implicit perceptual template promoting allocations of the same proportions as just witnessed. That is, when faced with a difficult distribution task, numerical matching might provide a suitable (low-level) strategy for young children helping them to guide their behavior.

From a wider developmental perspective, this account has some plausibility. It relates to work showing that for preschool children one-to-one matching strategies play a role in children’s development of counting (e.g., Gelman & Meck, 1983) and that they use one-to-one matching strategies also in other visual tasks (e.g., Collins & Laski, 2015). It seems to be an established domain-general strategy that helps young children to deal with different tasks and contexts. Given that this strategy can be exerted based on relatively simple or superficial perceptual features of the stimuli (Collins & Laski, 2015), we conceive of it as a rather perceptual strategy. This low-level perceptual strategy of numeric matching seems largely important when other strategies to guide the individual’s behavior are not available and it seems therefore unlikely that adolescents’ and adults’ preferences for the rich would be substantively impacted by that process.

Indirect evidence comes from studies demonstrating that younger preschool children rely on simple strategies (e.g., one-to-one correspondence) before appreciating more complex distribution strategies in sharing situations (Frydman & Bryant, 1988). Moreover, recent work demonstrates that the development of fair sharing is related to children’s numeric cognition (Chernyak, Harris, & Cordes, 2019). Direct evidence for a numerical matching account comes from a recent study by Essler et al. (2020). Three- to 5-year-old children who gave more to the rich and less to the poor recipient often did so by numerically matching the poor’s endowment rather than allocating him no resources. In addition, when asked to justify their allocation decisions, inequitable allocators often referenced the pre-existing numeric inequality. This could indicate that they used the differing amounts of possessions as a cue and potentially a justification for their own allocation behavior. Yet, this account falls short in explaining the effect of higher-order cognitive and affective mechanisms that lead to substantial variability in children’s allocations. Notably, while a few studies tried to rule out an effect of perceptual preferences in older children’s inequity aversion (e.g., Shaw & Olson, 2012), such controls have not been implemented in studies on children’s unequal resource allocations and the impact of numerical matching needs thus further examination.

One could speculate that children resort to this strategy when no other more dominant or suitable strategies are not present. That is, it should be primarily found in young children who do not have acquired other cognitive strategies or principle of resource distribution. Indirect evidence comes from a study that found links between lower cognitive abilities and an increased generosity towards a rich other in children with ASD (Paulus & Rosal-Grifoll, 2017). Moreover, given its perceptual nature, this strategy should break down if the types of resources to be distributed is rather different from the recipients’ initial endowment.

### Normative model

A fourth account takes as its point of departure the growing interest in young children’s understanding that social activities are regulated by social norms (Rakoczy & Schmidt, 2013). Likewise, a long tradition in developmental psychology has highlighted that young children develop a differentiated view on the nature of human norms (Smetana, 2013, Turiel, 1983). Recent studies have shown that preschool children endorse a variety of fairness norms from third parties and demand particular ways of resource distributions (e.g., Rakoczy et al., 2016, Rossano et al., 2011). While most studies focused on the early ontogeny of norms that relate to widely accepted moral standards (e.g., equal sharing), one needs to be aware that there are also normative beliefs that justify the perpetuation of inequality (cf. Starmans et al., 2017). There are at least two ways how young children could come to see an unequal distribution as a norm. On the one hand, young children might pick up these normative practices from the social environment (e.g., Dahl & Campos, 2013). For example, they might overhear normative talk in which adults accept and justify the current state (cf. Starmans et al., 2017). On the other hand, children might infer norms from a pre-existing inequality. Indeed, current work yielded evidence for such a “normativity of the factual” in young children (e.g., Roberts et al., 2017, Roberts et al., 2018). Thus, based on the observation that a rich individual possesses more resources than a poor individual, young children might construct a norm that holds that one recipient should have more resources than the other recipient. Thus, young preschoolers’ tendency to allocate inequitably might be seen as a normative concern preceding equality considerations that emerge later in development.

From a broader point of view, it should be noted that this account relates to an increasing interest in the emergence and structure of normativity in early childhood. A growing set of recent studies revealed that preschool children hold normative views on a variety of human behaviors such as game rules (Rakoczy, Warneken, & Tomasello, 2008), object use (Kenward, 2012), tool-use (Schmidt, Butler, Heinz, & Tomasello, 2016), and resource allocation principles (Wörle & Paulus, 2018). These norms are quickly acquired, sometimes from merely observing one single action (Schmidt, Svetlova, Johe, & Tomasello, 2016) or the status quo (Roberts et al., 2017, Roberts et al., 2018), and children enforce them even from third parties. Interestingly, it has also been shown that young children accept many reasons for inequality as being acceptable, even a simple statement that a recipient wants to have more than the other (Schmidt et al., 2016).

To date, no study has directly tested this possibility, but the results of recent studies provide some first empirical evidence. In the study by Essler et al. (2020), inequitable allocators judged inequitable allocations as fairer than equal and equitable allocations. That is, these children prioritized inequitable allocations over classical fairness principles. Moreover, in a study by Wörle and Paulus (2018), there were some 3-year-old children who protested against an equitable resource allocation (that is, giving more to a poor other than to a rich other) and affirmed an unequitable allocation. Although inconclusive, these results open up the possibility that some young children might have a norm to allocate more resources to a rich other. Given the recent findings on how quickly children infer the presence of norms (Schmidt et al., 2016) and move from an *is* to an *ought* to (Roberts et al., 2018), this approach is promising. In addition, this account offers a clear life-span perspective. For example, social psychological research has shown that adults’ belief in a just world relates to their justification of the status quo (Lerner, 1980). More specifically, the just world belief hereby refers to a cognitive bias justifying the status-quo (e.g., wealth and poverty) by interpreting differential wealth as the fitting consequence of individuals’ behavior. Thus, adults who believe in a just world might prefer the rich over the poor (and might hold a norm that everyone should do so), as the rich are justified in their advantaged positions by their more adequate or productive actions.

This model allows for the hypothesis that if children were to construct a social norm from the observation of preexisting inequalities, one should not only find a behavioral inclination to give more to rich than to poor, but also an explicit normative view and potentially an enforcement of this norm from third parties. This hypothesis has not been systematically explored as previous studies, in their attempt to answer different questions, have confounded this with other factors (e.g., giving to rich by means of taking from poor; Essler & Paulus, 2020).

## Open avenues and conclusion

The previous sections presented several theoretical models that account for recent findings on young children’s perpetuation of inequality. In this final section, we aim to integrate the discussion and point to avenues for future research.

We identified potential mechanisms that could underlie children’s preferential sharing with rich others. While the previous paragraphs presented each mechanism independently, it is possible that the phenomenon is not exclusively explained by one process, but an interplay of some of these processes. A speculative idea is that young children’s preferential sharing with the rich other might begin with an affective preference (potentially supported by simple numerical matching strategies) and is then superseded by strategic considerations. That is, the different processes might emerge at different developmental timelines and their relative impact might change across development. Indeed, studies on children’s reasoning about poor and rich others are suggestive for such a change: younger children’s favored evaluation of the rich other seem to be driven by an affective tagging mechanism based on the good outcomes, whereas older children reason about personal qualities such as effort, choice and ability (Sigelman, 2012, Sigelman, 2013). In a similar vein, adolescents increasingly see inequalities as justified when they pertain to individual achievements or efficiency considerations (Almås, Cappelen, Sørensen, & Tungodden, 2010). It should be noted that the increasing use of explanations in terms of free choice seems to relate to a derogation of poor others (Karniol, 1985). Next to developmental changes in the relative impact of different mechanisms, it is possible that one process could be subordinate to another process. For example, even though the numerical matching model is able to explain perpetuation of inequality on its own, the numerical matching strategy could also be employed in the service of a normative view that everyone’s endowment should be matched. Overall, a clarification of the impact of each proposed mechanism requires a consideration of its potential dynamic interplay with other mechanisms, and potential developmental changes in its relative impact.

Moreover, the review highlighted some inconsistencies in the findings that warrant further investigations. For example, on the one hand young children are less likely to favor the rich when they do not expect reciprocal behavior in the future (Kenward et al., 2015), on the other hand they are more likely to favor the rich when they have forgotten who was the rich (Li et al., 2014). It seems difficult to reconcile these results at the first glimpse. The debate would profit from replications of these findings as well as a closer empirical examination of the role of cognitive representations of the rich other. How exactly is the rich recipient represented and how do alterations in the cognitive representations affect children’s sharing?

What is the overall developmental picture on perpetuation or remedy of inequality that is suggested by the current literature? While recent work indicates that very young children share more with rich others than with poor others (Essler et al., 2020), this tendency declines in the course of the preschool years in favor of a concern for equality or equity (Charafeddine et al., 2016, Kenward et al., 2015, Wörle and Paulus, 2018). That is, young children increasingly adopt a strategy of equal sharing or a strategy for rectifying inequality (e.g., Elenbaas & Killen, 2016). Yet, at the same time, older children are also more likely to explain differences in wealth by reference to ability or effort (Sigelman, 2013), thus relying on an “equity” explanation to account for inequality (Leahy, 1983, Leahy, 1990). In other words, the development of intuitive theories that a rich person worked more or harder might lead to a preference for rich others. In this sense, a concern for equity can give rise to the perpetuation of inequality. At first sight, this seems to imply a U-shaped developmental pattern across early and middle childhood in which a tendency to perpetuate inequality is present in early development, seems to decline in the course of early childhood based on a strong concern for strict equality, and then seems to become stronger again in late childhood – potentially based on the developing notion that people possess stable personality traits (such as being diligent or lazy). These are then used to evaluate self and others, and to account for the existence of inequalities (Leahy, 1990). In line with Leahy (1990) this can be interpreted as a side-effect of children’s cognitive development and a tendency to interpret human behavior in terms of personality differences. In the transition to adulthood, the explanations of inequality then become potentially increasingly multidimensional including structural and systemic factors that could account for inequality (Flanagan et al., 2014).

Yet, it is unclear to which extent this pattern might be caused by different experimental tasks and the exclusive reliance on cross-sectional designs. Longitudinal studies could help to shed some clarity on this issue. Using comparable tasks across age groups would allow to reveal developmental changes. Moreover, it could clarify to which extent the psychological mechanisms underlying a potential preference for rich others change during development. In addition, the inclusion of several measures of young children’s appreciation of resource allocation principles could clarify whether young children simultaneously consider equality while perpetuating inequality – applying both principles to different targets or groups as implicated by Kohlberg (1986) – or whether there is indeed a clear developmental shift from perpetuation of inequality to an endorsement of equality.

A related point concerns the impact of the task. On the one hand, children’s perpetuation of inequality is not restricted to one particular type of resource allocation task, but seems to be evident across different tasks. Most notably, it has been reported in first-person sharing tasks (e.g., Kenward et al., 2015) in which children decide about resources that they could obtain themselves, and also in third-person distribution tasks (e.g., Essler et al., 2020, Li et al., 2014) in which children allocate resources between other recipients without receiving anything themselves. Moreover, a similar trend has also been observed in an instrumental helping task in which young children returned objects to helpees (Paulus, 2020). Nonetheless, all the tasks are characterized by children distributing similar resources to the ones that constitute the preexisting inequality of the different recipients. It would be highly interesting to see whether young children’s preference for the rich other is also evident in different types of prosocial behavior (for review see Paulus, 2018), for example in comforting. Indeed, assessing whether young children preferentially comfort rich over poor others might provide an informative test case for the four accounts. Most notably, if children’s behavior towards the rich other is mainly driven by an affective preference, one would hypothesize that they preferentially comfort the rich other. Yet, given that comforting behavior cannot be matched to resources, no such preference would be predicted by the numerical matching model.

Next to further experimental investigations, it would also be valuable to explore the ecological validity of the accounts and, most importantly, the (strength of the) presence of a preference for rich others in children’s everyday interactions and their lived experiences (cf. Rogoff, Dahl, & Callanan, 2018). Most notable, in which contexts is it present in children’s interactions with peers, and how does it affect children’s social relationships? In addition, what does it mean for a poor child to experience that her peers might be preferentially drawn towards a rich peer? While this question clearly goes beyond the scope of this review paper, it underlines the societal relevance of the topic.

Another point concerns the tension between children’s perpetuation of inequality and their emerging moral self-concept. That is, a majority of people value fairness and care about the well-being of others (Dawes et al., 2007). Most likely, they have a positive moral identity and view themselves accordingly (cf. Hardy & Carlo, 2011). Importantly, the moral self-concept emerges in the preschool years (Krettenauer, 2013). It relates to children’s own behavioral tendencies (Sengsavang & Krettenauer, 2015) and reflects children’s conscience development (Kochanska, Koenig, Barry, Kim, & Yoon, 2010). It would be interesting to explore whether children who perpetuate inequality nevertheless regard themselves as being fair or whether a moral self-concept is not yet developed.

Finally, previous work shows that older preschoolers’ tendency to rectify inequalities might be culture-specific. More precisely, it has been argued that in collectivist cultures children might prefer equal distributions in order to maintain relationships (Chai & He, 2017). One could thus wonder whether and to which extent a preference to allocate more to rich others (as so far only revealed in studies with Western children) might be culture-dependent or an universal characteristic of sharing behavior in young children. One could speculate that the four theoretical accounts outlined above would made different predictions for the existence of cultural and societal differences. Cross-cultural developmental research could thus constitute a further empirical touchstone for theories on young children’s tendency to perpetuate inequality.

### Conclusion

Taken together, recent research has repeatedly shown that a young preschool children tend to perpetuate inequalities by allocating more resources to rich others than to poor others. This is in contrast to the well-documented preference for equal sharing or equity in older children. While there is considerable research on the development of resource allocation behavior in older preschool children, we need more research to explore the early developmental origins of sharing behavior.
